# Evaluating predictive models for hemorrhagic transformation post-mechanical thrombectomy in acute ischemic stroke

**DOI:** 10.3389/fneur.2025.1549057

**Published:** 2025-06-25

**Authors:** Jiaxuan Wang, Jianyou You, Hui Yang, Zhongbin Xia, Xiangbin Wu, Moxin Wu, Xiaoping Yin, Zhiying Chen

**Affiliations:** ^1^Department of Neurology, Affiliated Hospital of Jiujiang University, Jiujiang, Jiangxi, China; ^2^Jiujiang Clinical Precision Medicine Research Center, Jiujiang, Jiangxi, China; ^3^Department of Medical Laboratory, Affiliated Hospital of Jiujiang University, Jiujiang, Jiangxi, China

**Keywords:** acute ischemic stroke, mechanical thrombectomy, hemorrhagic transformation, symptomatic intracranial hemorrhage, predictive methods

## Abstract

Acute ischemic stroke (AIS), a condition defined by a decrease in cerebral blood flow, is primarily treated through mechanical thrombectomy (MT) for blockages in major anterior circulation arteries. Approaches encompass stent retrieval, suction thrombectomy, or a combination of both. MT is increasingly recognized for its rapid revascularization, low hemorrhagic transformation (HT) rate, and extended therapeutic time window. Nonetheless, multiple risk factors lead to post-MT HT through different mechanisms, resulting in adverse outcomes such as increased mortality and morbidity. Therefore, assessing the relevant risks based on predictive models for post-MT HT is necessary. These predictive models incorporate a series of risk factors and conduct statistical analyses to generate corresponding assessment scales, which are then used to evaluate the risk of postoperative bleeding. As this is a rapidly developing field, there is still controversy over which model is more effective than another in improving clinical efficacy, and there is a lack of consensus on the comparison of these data. In this paper, we assess the accuracy of these predictive models using receiver operating characteristic (ROC) curves and the concordance C-index. Determining the most accurate predictive model for post-MT HT is crucial for improving the prediction of patient outcomes and for the development of tailored treatment plans, thereby enhancing clinical relevance and applicability.

## 1 Introduction

**Figure 1:**
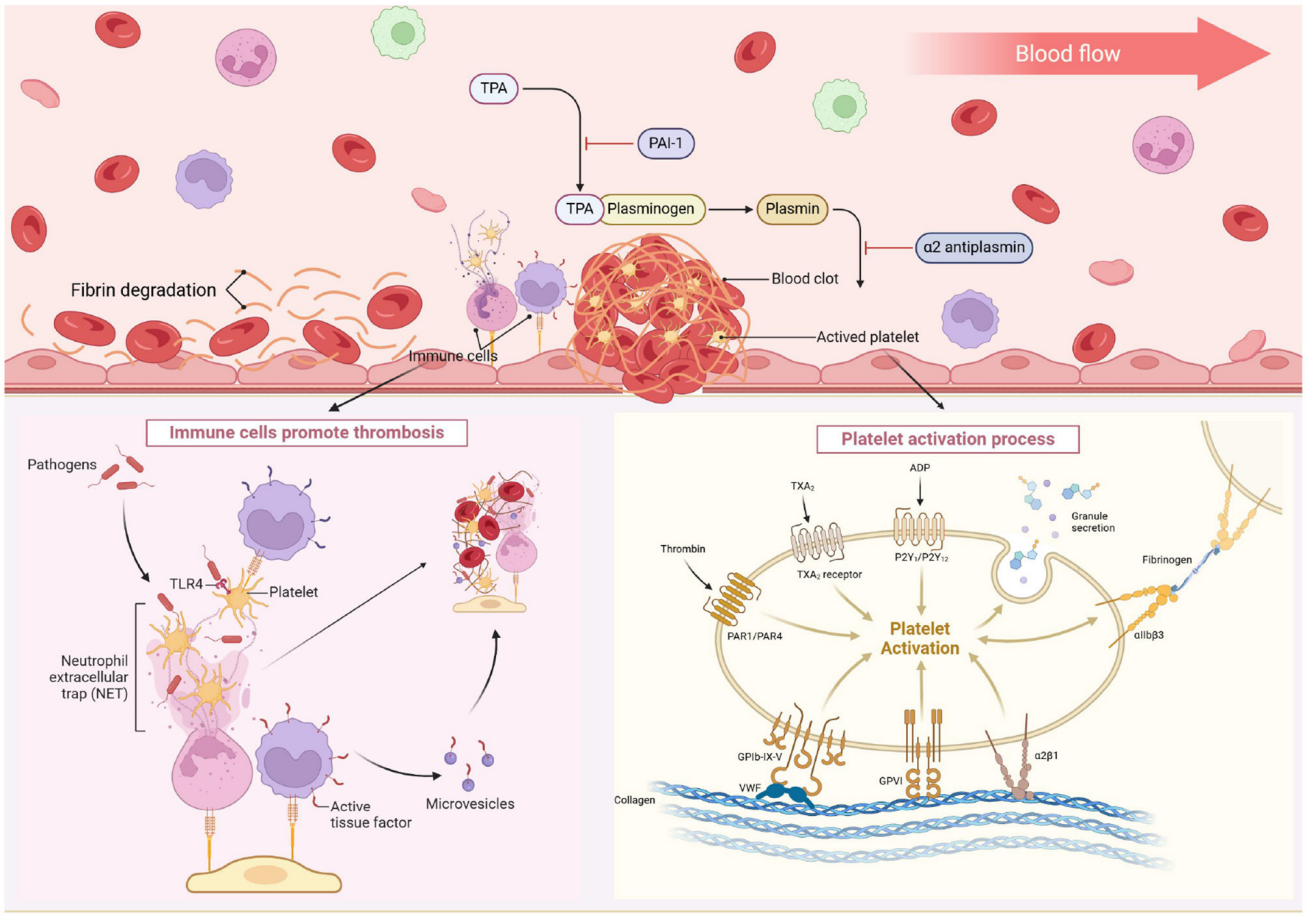
After the endothelial injury, exposed collagen triggers platelet adhesion to the vessel wall. These activated platelets release ADP and TXA2, leading to platelet aggregation and plug formation. They also release thrombin, which converts fibrinogen to fibrin. The injured vessel introduces tissue factors, starting a coagulation cascade that activates several coagulation factors and results in fibrin formation. Immune cells like neutrophils and monocytes play a role, with neutrophils releasing NETs and monocytes aiding in RBC recruitment and fibrin formation. TXA2, thromboxane A2; ADP, adenosine diphosphate; PAR, protease-activated receptor; GP, glycoprotein; VWF, von Willebrand factor; NET, neutrophil extracellular trap; RBC, red blood cell; TLR, toll-like receptor; PSGL-1, P-selectin glycoprotein ligand-1. Created with biorender.

Stroke is the leading cause of death and disability worldwide (1). Acute ischemic stroke (AIS) is the predominant type of stroke, constituting more than 80% of all strokes (2). AIS is characterized by an acute episode marked by the occlusion of arterial vessels that supply blood to the brain tissue (3). The onset of the disease results in ischemic blood flow and oxygen deprivation to the brain tissues, manifesting as neurological deficit symptoms (4). Thrombosis is a common underlying mechanism of AIS, and the blockage of cerebral blood flow leads to a series of pathophysiological changes (5) and is also a major cause of morbidity and mortality (6). Effective thrombus management is central to stroke treatment, and advances in thrombus research are helping to improve the efficacy of AIS therapy (7). Studies have shown that the process of thrombus formation involves platelet and coagulation factor pathways (8). After endothelial injury, exposed collagen promotes platelet activation and adhesion to the vessel wall (8), releasing adenosine diphosphate (forming platelet thrombi) (9) and thromboxane A2 (further promoting aggregation) (10). Concurrently, thrombin converts fibrinogen into fibrin (11), while tissue factor from injured vessels activates coagulation factors (FVII, FX, FIX, FII), resulting in fibrin formation (12). In addition, immune mediators are involved, with neutrophils releasing neutrophil extracellular traps and monocytes contributing to red blood cell (RBC) recruitment and fibrin formation (13) (Figure 1). Research shows thrombi have a dense outer shell of fibrin, vascular hemophilic factor, and platelets, with an inner core of erythrocytes, fibrin fibers, and platelets (14). The heterogeneous nature of thrombi is also confirmed by Senna's work (15). Thrombus removal via thrombolysis or mechanical thrombectomy (MT) is limited by factors like time window and thrombus composition (16). RBC-rich thrombi, with low viscosity and high RBC/platelet concentrations (17–20), are more prone to migration (21). Rapidly restoring cerebral blood flow is critical for focal cerebral ischemia. New experimental discoveries, such as targeting pyruvate kinase M2 (22) and using the elastase inhibitor peptide ShSPI (derived from the venom of Scolopendra hainanum), show promise in improving AIS outcomes (23). However, these findings have not been validated by large randomized clinical controlled trials, and therefore thrombolysis remains the mainstay of current treatments, with MT being more widely used. Intravenous injection alteplase or tenecteplase within 4.5 h of AIS shows comparable thrombolytic efficacy and improves 3–6 month functional outcomes. However, due to the strict time window and relatively low recanalization rate, fewer than 5% of AIS patients currently benefit from this treatment (24–26). Emerging evidence indicates that endovascular thrombus extraction within 24 h post-AIS onset (3), i.e., MT, can significantly improve the rate of favorable prognosis and reduce disability. Anterior circulation large vessel occlusion (aLVO) is an important cause of AIS (27). Thereby obstructing blood flow to the anterior part of the brain and precipitating AIS. Numerous randomized clinical trials have established MT as the standard therapy for AIS due to aLVO, with a high reperfusion rate of up to 80% and advantages such as rapid reperfusion, low bleeding conversion rate, and an extended treatment time window (28–32). It is gradually becoming more widely used in clinical treatment.

**Figure 2:**
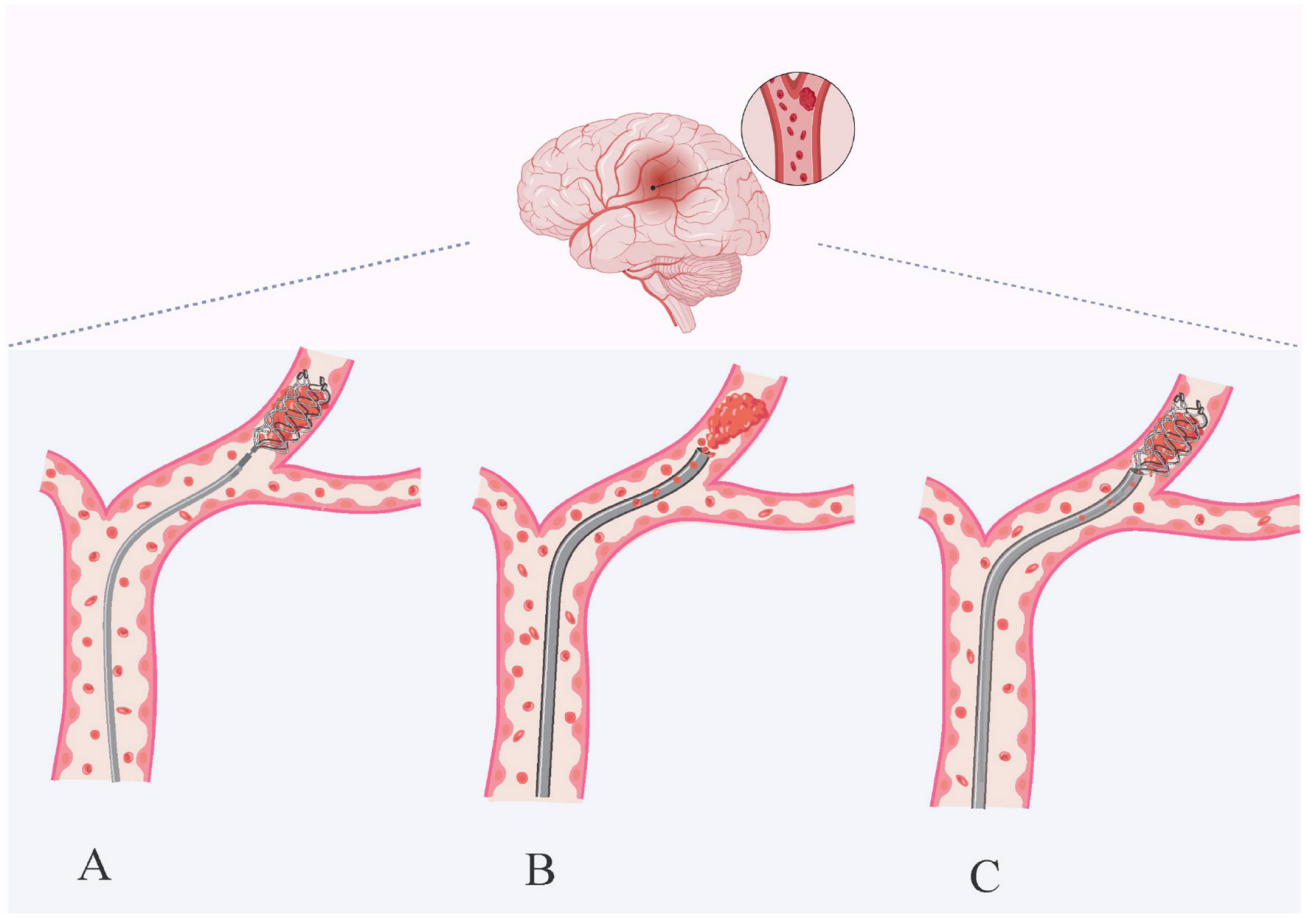
Techniques for mechanical thrombectomy. Stent retriever thrombectomy **(A)**, aspiration thrombectomy **(B)** and combined techniques **(C)**. Stent retriever thrombectomy uses a stent retriever to reach the occluded vessel, pass through the thrombus, which is fully integrated into the expanding stent, and then withdraw to remove it. Aspiration uses a large-bore catheter to contact the thrombus and slowly withdraws the catheter to suction out the thrombus under continuous negative pressure. The combined technique is a combination of stent retrieval and aspiration thrombectomy. Created with biorender.

Timely intervention in AIS involves opening the blocked blood vessels to salvage the ischemic brain tissue and penumbra. Prompt and effective recanalization is fundamental for a favorable prognosis in AIS patients. The optimal outcome of MT on aLVO is contingent upon rapid revascularization and avoidance of post-procedural complications (33). MT encompasses stent thrombectomy, aspiration thrombectomy, and their combined techniques. The use of balloon catheters can reduce the number of thrombectomy attempts, prevent distal embolization, and improve surgical outcomes (34). A stent thrombectomy device is a self-expanding metal mesh tube inserted percutaneously via a microcatheter, which encapsulates the thrombus and is then withdrawn to achieve reperfusion (3, 35, 36). Second-generation stents, such as Solitaire and Trevo, have become the primary treatment for acute stroke, offering high thrombus removal rates and low complication rates (37, 38). However, it requires a high level of technical proficiency, and the stent retriever thrombectomy technique carries risks such as endothelial damage and embolic escape (39). Thrombectomy techniques employ large-bore catheters to manually remove thrombi using suction pumps or syringes (35). The COMPASS trial demonstrated that aspiration thrombectomy yields functional outcomes comparable to stent thrombectomy after initial thrombus removal, with similar 90-day follow-up results (40). With the advent of large-bore distal suction catheters, aspiration may enhance efficacy (41). Although thrombus extraction can reduce the risk of thrombus escape, it is relatively less effective for harder or deeper thrombi (42). The Solumbra technique combines large-bore aspiration catheters with stent thrombectomy technology. It guides the aspiration catheter and stent to the thrombus site via a microguidewire, then releases the stent and connects it to a negative pressure device to enhance thrombectomy efficacy (43) (Figure 2). While stent-assisted suction thrombectomy increases the 24-h subarachnoid hemorrhage risk. Combined therapy has successfully improved recanalization rates (36). Meta-analyses confirm that combined approaches outperform stent thrombectomy alone in achieving vascular recanalization (44, 45). Patients with AIS treated with stents or distal suction catheters may experience damage to the vessel wall and intimal injury, as well as post-procedural risk of HT, and multiple risk factors are present. Post-treatment HT refers to HT occurring after MT and is a type of HT (46). Prospective studies of MT have demonstrated that an important risk factor for poor prognosis after MT is the occurrence of HT in the ischemic territory post-thrombectomy (47), which increases the risk of death and disability.

**Figure 3:**
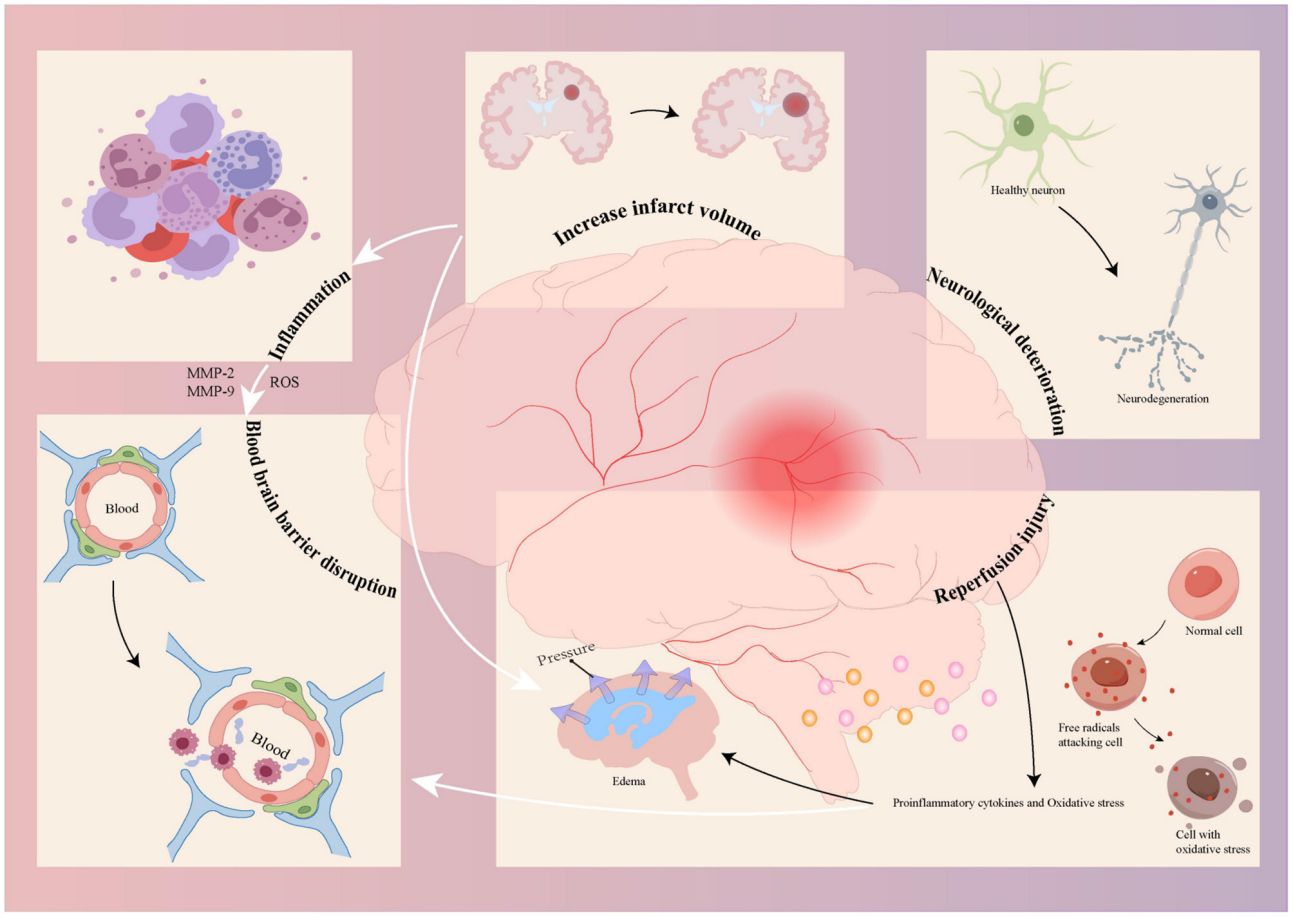
The mechanism of Hemorrhagic transformation related to thrombectomy includes the mechanism of Hemorrhagic transformation induced by thrombectomy and the pathological process of cerebral infarction itself. The use of drugs in the early stage of surgery and endovascular mechanical devices to remove thrombus can lead to reperfusion injury and damage to the blood-brain barrier, while the course of ischemic cerebral infarction itself includes inflammatory responses, neuronal cell death, and the expansion of the cerebral infarction volume. Different mechanisms can interact to cause more severe damage, Blood-brain barrier impairment is made worse by ROS, MMP-2, and MMP-9 produced by the inflammatory responses; large cerebral infarction brought on by an enlarging infarct size results in an inflammatory response and edema; and the cascade response brought on by reperfusion injury causes edema and aggravates blood-brain barrier impairment. ROS, reactive oxygen species; MMP, matrix metalloproteinase.

Patients with AIS undergoing thrombolysis or endovascular therapy may experience a severe complication known as HT (48). HT denotes bleeding phenomena caused by restoring perfusion in the ischemic area following AIS (49). The current diagnostic criteria for HT include: hemorrhage not evident on the initial cranial computed tomography (CT) or Magnetic Resonance Imaging (MRI) following a cerebral infarction, but subsequent scans reveal intracranial bleeding or hemorrhagic infarction (50, 51). Postoperative HT must be differentiated from contrast agent retention, in which hemorrhagic sites may occur either within or remote from the infarcted area (52, 53). A failure in promptly managing postoperative complications with HT leads to further brain tissue damage and an increased risk of adverse outcomes, including disability and death (54). However, HT is associated with multiple risk factors, including prolonged thrombolysis or endovascular treatment time, high preoperative blood pressure (BP) (systolic BP >180 mmHg and diastolic BP >100 mmHg), hypointense changes on head CT, stroke severity, and infarct size (55, 56). Additionally, age, hyperglycemia, prior use of antiplatelet drugs, and atrial fibrillation are also associated with an increased risk of HT (57). Blood biomarkers, including matrix metalloproteinase-9 (MMP-9), S100B, activated C-reactive protein, and genetic factors, have also been linked to the development of HT. These risk factors contribute to HT through mechanisms associated with thrombectomy. These mechanisms include factors inherent to the procedure itself, as well as the pathological processes inherent to cerebral infarction. Early thrombus removal, whether via pharmacological agents or endovascular mechanical devices, can lead to reperfusion injury and blood-brain barrier (BBB) dysfunction (58, 59). Cerebral infarction pathology encompasses ischemia-related inflammatory mechanisms, neurologic impairment, and infarct size (60–62). These mechanisms may interact and cause more severe damage. In the inflammatory response, activated immune cells produce reactive oxygen species and MMPs (MMP-9, MMP-2), culminating in BBB destruction (63). Reperfusion exacerbates oxidative stress, leading to excessive production of pro-inflammatory cytokines and triggering a pathological cascade that causes cerebral oedema and BBB disruption (64). Neurological dysfunction, a neuroinflammatory mechanism, may result in acute neuronal injury or death. Reperfusion injury exacerbates brain damage and neurological deficits, thereby influencing the occurrence of HT (65). Inflammatory responses and edema resulting from massive cerebral infarction increase the risk of HT (66) (Figure 3). Therefore, early and accurate prediction of postoperative HT risk is important. Numerous investigations have concentrated on identifying risk factors for postoperative HT in patients with AIS for potential intervention. Several scoring scales evaluate risk factors and can assist in predicting postoperative HT (Table 1). The modified Thrombolysis in Cerebral Infarction (TICI) score is used to assess reperfusion after MT, categorized into grades 0–3, where grade 0 indicates no reperfusion and grades 2b−3 indicate successful reperfusion (67). Collateral perfusion is critical for maintaining the ischaemic penumbra, and is classified using the American Society of Interventional and Therapeutic Neuroradiology/Interventional Radiology grading system, where grade 0 indicates no collateral blood flow and grade 4 indicates rapid blood flow (68). Preoperative infarct core area is positively correlated with HT occurrence (69). Its area is quantified using the Alberta Stroke Program Early CT Score (ASPECTS) (70), with lower scores indicating larger infarct areas. Baseline stroke severity is assessed using the National Institutes of Health Stroke Scale (NIHSS) score (71), with higher NIHSS scores associated with more severe hypoxic environments and BBB dysfunction (66, 72). Analyzing relevant factors through these methods to predict HT after MT and assess HT risk is of significant clinical importance for improving post-MT prognosis (73). A common complication of HT after surgery is symptomatic intracranial hemorrhage (sICH), which is associated with worsening neurological deficits (74–76), reducing the risk-benefit ratio of endovascular therapy. Current research suggests that the occurrence of sICH is strongly associated with the risk of adverse functional outcomes and patient death (77–79). Consequently, clinicians are seeking suitable indicators to predict sICH following mechanical thrombectomy to enhance the prognosis of patients with cerebral infarction. Due to the many risk factors associated with sICH and the limited predictive value of individual factors for sICH, it is challenging to develop a comprehensive postoperative sICH prediction method, and there is no accurate and recognized prediction method in clinical practice. Therefore, more accurate and simpler sICH prediction methods are needed to prevent the occurrence of unfavorable prognosis after MT and improve the survival rate and quality of life of patients.

**Table 1:** The application of rating scales.

**Rating scales**	**mTICI**	**ASITN/SIR**	**ASPECTS**	**NIHSS**
**Range**	0–3 (level 2 includes 2a, 2b, 2c)	0–4	0–10	0–42
**Result**	Grade 0 indicates no perfusion and grades 2b-3 are defined as successful reperfusion	Grades of 0–1 indicate poor collateral circulation and grades of 3–4 indicate good collateral circulation status	Points of 0 indicate the presence of severe ischemia, and points of 10 indicate a low likelihood of vascular occlusion	Points 0–1 tend to be normal, and 21 or more points indicate a severe stroke.
**Functions**	For assessment of postoperative distal flow reperfusion in occluded vessels	Assessing the status of the collateral circulation	Quantitative assessment of the ischemic region of the middle cerebral artery	Assessing the extent of neurologic deficits

mTICI: modified thrombolysis in cerebral infarction; ASITN/SIR: American Society of Interventional and Therapeutic Neuroradiology/Society of Interventional Radiology; ASPECTS: Alberta Stroke Programme Early CT Score; NIHSS: National Institutes of Health Stroke Scale.

## 2 Prediction of HT in cerebral infarction with MT

### 2.1 Existing forecasting methods

In this section, we selected studies with large sample sizes and a relatively long duration following the generation of the prediction scores for comparative analyses.

The TICI-ASPECTS-Glucose (TAG) score integrates blood glucose levels, ASPECTS, and the TICI score into a multivariate logistic regression analysis model, utilizing the odds ratios (OR) of the predictor variables to calculate a TAG score that aids in predicting sICH risk (80) and individual patient risk. The TAG score classifies sICH risk into three categories: a score of 0–2 for the low-risk group, 3–5 for the intermediate-risk group, and 6–7 for the high-risk group. The inclusion of additional sICH-related predictors is likely to enhance the model's robustness (81). This investigation encompassed a relatively large sample and externally validated the predictive score. Additionally, the components of the score are straightforward to assess and apply in clinical practice. This comparative analysis evaluated the blood glucose level, ASPECTS, TICI as single factors, and the TAG score for predicting sICH after MT, utilizing the area under the receiver operating characteristic curve (ROC-AUC). The findings indicated that in the derivation cohort, the TAG score ROC-AUC was 0.79, demonstrating higher sensitivity and specificity for predicting post-MT sICH, whereas the TICI univariate analysis predicted postoperative sICH with higher sensitivity, and the TAG score predicted with higher specificity. This discrepancy can be attributed to the derivation cohort's use of the European Cooperative Acute Stroke Study (ECASS) III definition of sICH (82), differing from the definition employed in the validation cohort. Consequently, the TAG score exhibited superior predictive power for sICH after MT when assessed using the ECASS III criteria. Nevertheless, results from both cohorts revealed an elevated risk of sICH associated with an increased TAG score.

The Italian Registry of Endovascular Stroke Treatment in Acute Stroke-Symptomatic Intracranial Hemorrhage (IER-SICH) nomogram defines sICH as any type of ICH in which the NIHSS score increased by ≥4 points from baseline or resulted in death within 24 h. The initial model incorporated NIHSS score, time from symptom onset to end of course, age, unsuccessful revascularization, and Careggi collateral circulation score as predictors of sICH, subsequent models expanded upon this by the first model by removing the Careggi collateral circulation score and adding the ASPECTS score to assess discriminatory performance using the ROC-AUC (83, 84). The IER-SICH nomogram represents the inaugural model developed and validated for the prediction of sICH after MT. The prediction model was developed utilizing a cohort of patients receiving bridging therapy and validated it using a cohort of patients undergoing MT (84). However, studies confirmed that the incidence of sICH in patients receiving bridging therapy was similar to MT (85). Within the training cohort, the first model of the IER-SICH nomogram had a ROC-AUC of 0.778 and the second predictive model had a ROC-AUC of 0.733, indicating that the first model had better predictive power than the second model. The Careggi collateral circulation score and the ASPECTS score correlate significantly, as demonstrated by their correlation (86). The ASPECTS score enjoys broader application and serves as an exclusion criterion in randomized controlled MT trials (29, 30). Similarly, within the test cohort, the first model achieved a ROC-AUC of 0.709, and the second prediction model achieved a ROC-AUC of 0.685, which indicated that the training set exhibited greater predictive accuracy, given that the training group was comprised of patients receiving bridging therapy. The test cohort consisted of patients undergoing MT. The study further established that the incidence of sICH was equivalent in both scenarios.

The ASIAN score utilizes the Heidelberg bleeding Classification of Hemorrhage (87), and 24 h post-MT, sICH diagnosis required the presence of new intracranial hemorrhage, associated with an increase in NIHSS score of >4 points from the pre-deterioration level or an increase of >2 points in any classification of intracranial hemorrhage, or in cases of neurologic deterioration necessitating intubation, endocraniectomy, placement of an external ventricular drain, or other significant medical or surgical interventions. The ASIAN score encompasses the ASPECTS score, collateral circulation status, baseline blood glucose level, number of embolectomy device passes, and time from symptom onset to groin puncture as independent predictors of sICH (88), utilized for predicting sICH post-MT in patients with AIS. An increased risk of sICH corresponded with higher ASIAN scores. The ASIAN score was evaluated using a risk model with a C-index and a calibration curve, which were used to assess the discriminatory and calibration power of the risk model. In this study, ROC curves were constructed, and the C-index was used to compare the predictive accuracy of the TAG score, the IER-SICH nomogram, and the ASIAN score for the prediction of post-MT sICH in an Asian population. The results indicated that the ASIAN score exhibited better discriminatory power than the TAG score and the IER-SICH nomogram in both the derivation and validation cohorts, and the prediction of postoperative sICH demonstrated better sensitivity and specificity. Given that the patient selection criteria in this score are Chinese, it is more suitable for predicting post-MT sICH in Asian populations.

### 2.2 Emerging prediction methods

We have summarized and evaluated risk scores that have been recently developed, including the incorporation of new predictors, to select predictive scores that are more applicable to patients.

The Time-ASPECTS-Glycemia-EVF (TAGE) score, which defines sICH as being any intracranial hemorrhage accompanied by neurologic deterioration (an increase of ≥4 points in the NIHSS score from baseline) on imaging 24 h post-MT, with early venous filling (EVF) included as a predictor. EVF, a potential imaging biomarker, is readily identifiable on digital subtraction angiography following successful MT recanalization (89–92), and serves as a strong predictor of sICH after MT (89, 90). The TAGE score includes predictors, including a prolonged delay from onset to successful recanalization, low ASPECTS, hyperglycemia on admission, and the presence of post-MT EVF, assessed for calibration with the Hoer-Lemeshow test and discrimination with ROC-AUC (93). The risk of sICH escalates with increasing TAGE scores. The ROC-AUC values were 0.832 for the derivation cohort and 0.801 for the validation cohort, suggesting that the TAGE score's discriminatory power was superior in both cohorts, likely attributed to the substantial sample size of patients successfully treated with the predictive method of Janvier et al. in the derivation and validation cohorts. Comparing the TAGE score with the TAG score, the TAGE score demonstrates superior discrimination, as it utilizes variables that can be rapidly ascertained under realistic conditions of acute stroke treatment. However, the TAGE score's smaller sample size relative to the TAG score and the necessity of diagnosing EVF post-therapeutic decision severely limit its influence on therapeutic decision-making. The TAGE score represents the inaugural practical tool to incorporate EVF results into sICH prediction.

The Systolic BP-Time-Blood Glucose-ASPECTS (STBA) score integrates time from systolic blood pressure on admission, time from AIS onset to groin puncture, blood glucose, and ASPECTS score on admission as predictors to create a more feasible scoring system, predicting an increased risk for sICH with higher scores (76). The STBA score had a ROC-AUC of 0.858 in the derivation cohort, and similar sensitivity and specificity to the TAG score, although the study data source was Chinese patients, and the sample size was small. The ACTS model, utilizing ASPECTS, collateral circulation status, Trial of Org10172 in Acute Stroke Treatment (TOAST) classification and serum glucose, is a rapid and easy-to-implement prediction model for preoperative evaluation to predict the risk of sICH (94). The score demonstrated robust predictive power with an ROC-AUC of 0.797 in the derivation cohort and 0.727 in the validation cohort. However, the study's inclusion criterion of patients treated within 6 h of symptom onset limits its scope, and may offer significant predictive value for the selected patient group, yet its applicability to the broader AIS population undergoing MT is uncertain. Chang et al. collected data on aLVO patients with contrast enhancement (CE) on CT of brain planarization following MT. Utilizing ASPECTS, they estimated the extent and location of CE, identifying two predictive factors: CE-ASPECTS and the distribution location of CE, encompassing the internal capsule area and the middle cerebral regions. The CE-Age-Glucose-Atrial Fibrillation (CAGA) score (95) incorporates the four aforementioned variables. The score employs the discriminatory power of the ROC-AUC assessment, which, according to ECASS II, defines sICH as hemorrhage associated with an increase in NIHSS score of ≥4 points on follow-up brain examination. This comparative analysis revealed the CAGA score in comparison with the TAG score, demonstrating that the CAGA score's ROC-AUC was 0.853, with high sensitivity and specificity. The CAGA score represents the inaugural predictive score developed for sICH risk assessment in patients with CE. However, this score's study is limited by a small sample size, and utilizing a test of independence is inappropriate, as it is susceptible to bias, resulting in potentially imprecise results.

### 2.3 Comparison of prediction methods

**Figure 4:**
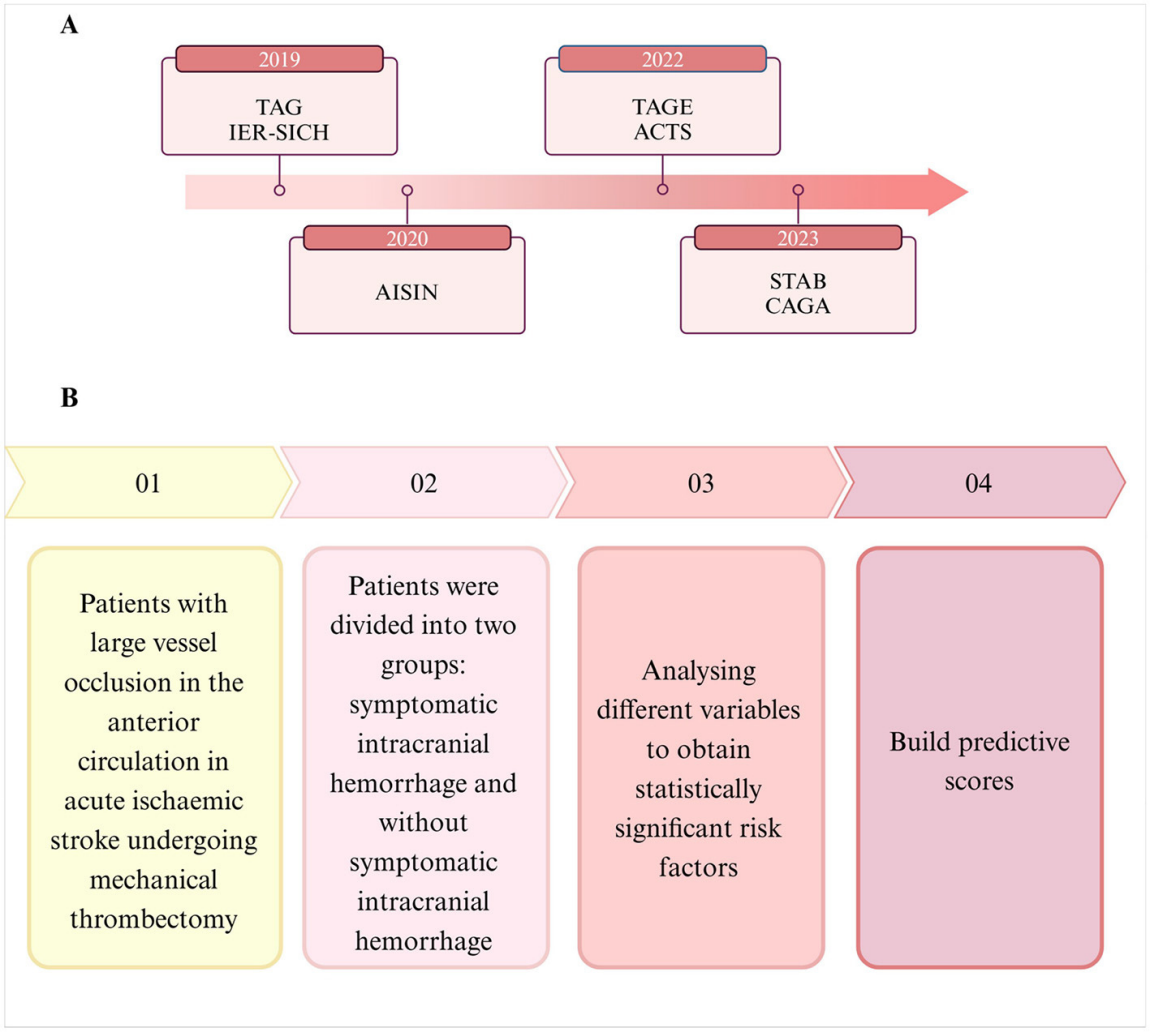
**(A)** We retrieved predictive scores for hemorrhage transformation after mechanical thrombectomy for acute ischemic stroke for the last 5 years, and collated and analyzed them by year. **(B)** We describe the process of constructing the predictive scores. Created with biorender.

We categorize these prediction methods into pre-existing and emerging methods based on their temporal emergence and describe the process of their construction (Figure 4). In addition, we have summarized the advantages and disadvantages of the prediction methods, their clinical application value, and suggested optimization measures (Table 2). A comparison of the prediction scores revealed that the sensitivity of the STBA score is comparable with that of the TAG score, and the specificity is higher. Consequently, the ROC-AUC for the STBA score is higher, indicating better discrimination power, although the sample size is smaller than that of the TAG score. This may be because the STBA score incorporates more discriminative predictive variables, such as imaging features and laboratory indicators, which results in significantly higher specificity compared to the TAG score. Certain studies constructed ROC curves and utilized the C-index to evaluate the predictive accuracy of the ASIAN score, TAG score, and the IER-SICH nomogram. The sample size for TAG scoring is the smallest, while the IER-SICH histogram has the largest sample size and a relatively high C-index. Therefore, the IER-SICH results may be more stable and have greater statistical power. Its ROC-AUC is the smallest, possibly because the dependent variables it includes (such as age and blood pressure) have limited discriminatory power. The ASIAN score had a mid-range sample size, and the results indicated that it had the highest sensitivity and specificity, namely, its prediction accuracy was superior to that of the TAG score and IER-SICH nomogram. In summary, it can be concluded that the STBA score had the highest ROC-AUC and the best discriminatory power among the scores. Furthermore, the ASIAN score exhibited higher sensitivity and specificity and better predictive accuracy than other scores. The ASIAN score was developed and validated for Chinese patients and is more applicable to the Asian race, which has a higher incidence of sICH. Therefore, for ASIAN AIS patients, using the ASIAN score to predict the occurrence of sICH after MT is a more appropriate choice.

**Table 2:** Comparison of methods for predicting hemorrhagic transformation from mechanical thrombectomy.

**Score**	**Items**	**Evaluation indicators**	**Sample**	**advantages**	**Limitations**
**TAG** **(****80****)**	Glucose	ROC-AUC: 0.790 C index: 0.680	578	Predictors are easy to detect	Lower discriminatory performance
	ASPECTS score				
	TICI score				
**IER-SICH** **(****84****)**	NIHSS score	ROC-AUC: first model, 0.778 second model, 0.733 C index: 0.710	988	With the largest sample size, it is the first predictive model developed for predicting HT after MT	The training set selection criteria were patients receiving bridging therapy
	Onset-to-end procedure time				
	Age				
	Unsuccessful recanalization				
	Careggi collateral score				
	ASPECTS score				
**ASIAN** **(****88****)**	ASPECTS score	C index: 0.771	629	More suitable for Asians; higher Prediction accuracy than TAG and IER-SICH	Collecting data retrospectively may lead to systematic bias in the outcomes of a study
	Baseline glucose level				
	Poor collateral circulation				
	Passes with retriever				
	Onset-to-groin puncture time				
**TAGE** **(****93****)**	Time-to-successful reperfusion	ROC-AUC: 0.832	402	TAGE score is the first utility to incorporate EVF results into sICH predictors	EVF cannot be detected until reperfusion is done and treatment decisions are made
	ASPECTS score				
	Glucose level				
	Early venous filling				
**STBA** **(****76****)**	Systolic BP	ROC-AUC: 0.858	268	The strongest discriminatory performance	The sample size is relatively small
	Time from onset to groin puncture				
	Blood glucose				
	ASPECT score				
**ACTS** **(****94****)**	Collateral circulation status	ROC-AUC: 0.797	433	Higher incidence of sICH; predictors were available preoperatively	Strict inclusion criteria: patients within 6 h of symptom onset
	Baseline ASPECTS				
	TOAST type				
	Serum glucose				
**CAGA** **(****95****)**	CE-ASPECTS	ROC-AUC: 0.853	109	The first predictive score for risk of sICH occurrence in patients with CE	The sample size was relatively small and no test of independence was performed
	CE locations				
	Age				
	Atrial fibrillation				
	Serum glucose				

TAG, TICI-ASPECTS-Glucose; ASPECTS, Alberta Stroke Programme Early CT Score; TICI, Thrombolysis in Cerebral Infarction; ROC-AUC, The Area Under The Receiver Operating Characteristic Curve; IER-SICH, Italian Registry of Endovascular Stroke Treatment in Acute Stroke-Symptomatic Intracranial Hemorrhage; NIHSS, National Institutes of Health Stroke Scale; MT, Mechanical Thrombectomy; HT, Hemorrhagic Transformation; sICH, Symptomatic Intracranial Hemorrhage; TAGE, Time-ASPECTS-Glycemia-EVF; EVF, Early Venous Filling; STBA, Systolic BP-Time-Blood Glucose-ASPECTS; BP, Blood Pressure; ACTS, ASPECTS-Collateral Circulation Status-TOAST-Serum Glucose; TOAST, Trial of Org10172 in Acute Stroke Treatment; CAGA, CE-Age-Glucose-Atrial Fibrillation; CE, Contrast Enhancement.

## 3 Evaluation of the clinical utility of prediction methods

Early and timely consultation of patients with AIS is the best measure to improve quality of life and save lives (96). However, prehospital delays, low numbers of neurointerventionists, limited resources, patient awareness, health literacy, adherence to stroke prevention, government policies, insurance reimbursement systems, and stroke advocacy in professional organizations can affect timely consultation and lead to a poorer prognosis (97). Similarly, patients with AIS undergoing MT are prone to adverse events such as HT, and the use of predictive methods to assess HT risk can minimize its incidence and improve the prognosis. The nomogram prediction method constitutes a vital component in modern medical decision-making, enabling better individualized disease-related risk assessment, and is widely used in oncology, surgery, and other areas (98, 99). Risk scores assist patients in making treatment decisions (100) and improve outcomes in patients with poor prognoses. Most of the existing methods for predicting post-MT HT analyze data across various ethnic groups. When using TAG and IER-SICH scores in non-Western populations or with small sample sizes, caution should be exercised. While these scores can serve as preliminary screening tools and assist in clinical decision-making, they should always be used in conjunction with other assessment indicators to facilitate comprehensive decision-making. Specifically for Asians, fewer predictive methods for post-MT HT exist. For Asian AIS patients, the ASIAN score demonstrates greater suitability in predicting post-thrombolysis sICH risk. According to previous studies, the incidence of post-MT HT is elevated among Asians (78). Therefore, additional studies collecting data from Asian ethnic groups are necessary to develop more specific prediction methods to provide them with more appropriate treatment options. However, only a limited number of risk-scoring systems have been developed and utilized specifically for predicting post-MT HT. There are more risk-scoring models for the assessment of HT after intravenous thrombolysis; however, it remains uncertain whether these models are applicable to predicting post-MT HT (101). Owing to the paucity of data on predictors of HT after MT, particularly sICH, the findings of relevant studies are inconsistent. These inconsistencies primarily stem from the lack of external validation, relatively small sample sizes, and the low incidence of sICH (102, 103). Thus, these scoring models should be combined with risk stratification tools, ancillary evaluations, and clinician judgment to guide holistic patient management. The independent risk factors encompassed by current methods for predicting post-MT HT represent only a subset, with other potential risk factors remaining unexplored. It is imperative to collect more relevant data, optimize the experimental design, increase the sample size, perform external validation, and conduct independent analyses for different populations to develop targeted prediction methods. Furthermore, efforts should be made to improve clinical applicability, including dynamic monitoring strategies and model simplification. Early prediction of HT after MT can provide an assessment of the risk associated with the disease, enabling physicians to take timely action to prevent further clinical deterioration and develop optimal treatment strategies (104, 105), and assist in the acute management of patients by aiding physicians, patients, and their families in making realistic prognostic decisions (101). In addition, HT prognostic methods can provide patients and their families with data to clarify the risks and benefits of treatment (80). They can also facilitate the early identification of high-risk HT patients, ensuring they receive intensive postoperative management, enhanced detection and treatment of hypertension and hyperglycemia, and the deferral of early antithrombotic therapy, among other interventions (84, 106). Post-MT HT is closely related to poor patient prognosis, leading to short- and long-term functional deterioration, and is a critical indicator for clinical management (107, 108). The accurate prediction of HT is crucial for guiding the precise treatment of AIS (109). When the risk of HT, particularly sICH, is elevated, the risks associated with HT, as well as the benefits and drawbacks of MT, must be meticulously assessed (110) to determine therapeutic strategies aimed at enhancing the safety of MT and improving patient prognosis (111, 112).

## 4 Conclusions and outlook

MT is a critical treatment for patients with AIS due to aLVO, and postoperative HT, particularly sICH poses a significant threat to patients' lives. Current methods for predicting HT post-MT are particularly well-suited for sICH prediction, and among these, the ASIAN score offers distinct advantages. The efficacy of current prediction methods is constrained by an incomplete analysis of risk factors, resulting in significant variability in clinical and laboratory data across diverse patient populations. By aggregating heterogeneous data from various groups through meta-analysis, we can derive comprehensive predictors that are broadly applicable. Moving forward, we can develop multimodal imaging, integrated with artificial intelligence and machine learning algorithms, to create more accurate and reliable predictive models. Furthermore, a more thorough analysis of reliable and valid independent risk factors is warranted, encompassing blood indices, DSA indices, time indices, and biomarkers, as well as genetic factors associated with bleeding risk. This approach aims to enhance personalized treatment and management of AIS patients through their proven validity and reproducibility, thereby improving the prediction of postoperative HT.
